# Virtual reality-based stress paradigms and early adversity: a scoping review of mechanisms and stress-response systems

**DOI:** 10.3389/fpsyt.2026.1881373

**Published:** 2026-07-17

**Authors:** Mara S. Singeap-Tiron, Cristiana Amalia Onita, Daniela Viorelia Matei, Petru Romeo Dobrin, Ioan Gotca, Mihaela-Alina Radeanu, Diana I. Petrescu-Miron, Veronica Mocanu

**Affiliations:** 1 Grigore T. Popa University of Medicine and Pharmacy Iasi, Iasi, Romania; 2 Institute of Psychiatry “Socola”, Iasi, Romania

**Keywords:** adverse childhood experiences, autonomic nervous system, early adversity, eating behavior, HPA axis, psychosocial stress, stress paradigms, virtual reality

## Abstract

**Background/Objectives:**

Virtual reality (VR)-based stress paradigms are increasingly used to induce stress in controlled yet ecologically valid settings. However, the diversity of VR-based stress paradigms and their differential engagement of stress-response systems remain insufficiently synthesized. This scoping review aimed to map VR stress paradigms and examine how they engage subjective, autonomic, endocrine, neural, and behavioral responses, while considering the role of early adversity.

**Methods:**

A structured literature search was conducted across Web of Science, ScienceDirect, SpringerLink, and Google Scholar for studies published between January 2012 and June 2026. Eligible studies used VR to induce stress and assessed psychological and/or physiological outcomes. After screening, 21 studies were included and grouped into clusters based on shared mechanisms and methodological features.

**Results:**

Five clusters were identified: (1) social-evaluative, (2) cognitive/performance-based, (3) threat/fear-based, (4) analogue trauma, and (5) complex ecologically valid paradigms, with implications for stress-related behavioral outcomes, including eating behavior. VR consistently elicited subjective and autonomic responses, while endocrine and neural responses varied by paradigm type. Social-evaluative paradigms showed robust subjective and autonomic activation with variable hypothalamic–pituitary–adrenal axis responses. Threat-based paradigms elicited strong autonomic and affective responses, whereas cognitive paradigms were associated with performance-related stress and limited endocrine activation. Evidence on early adversity suggested differential sensitivity to specific stressor types; however, only a subset of studies directly assessed adverse childhood experiences or childhood trauma, and its role as a moderator of stress reactivity remains underexplored.

**Conclusions:**

VR-based stress paradigms do not induce stress as a unitary construct but selectively engage distinct response systems depending on paradigm characteristics. Integrating early adversity may enhance the precision of VR-based stress research. This framework may support future investigations of stress-related behaviors, including eating behavior, in ecologically valid environments. It may also offer promising applications for personalized nutrition; however, these implications were not directly assessed in the included studies.

## 1 Introduction

Stress is a complex psychobiological process arising from the interaction between environmental demands and an individual’s perceived coping resources, involving coordinated activation of emotional, cognitive, physiological, and behavioral systems. Classical models conceptualize stress responses as driven by the interplay between the autonomic nervous system and the hypothalamic–pituitary–adrenal (HPA) axis, with downstream effects on cognition, decision-making, and health outcomes (1). Importantly, stress responses are not unitary but vary depending on the nature of the stressor and its psychological meaning (2).

Experimental research on acute stress has traditionally relied on standardized laboratory paradigms, most notably the Trier Social Stress Test (TSST), which provides a robust and reproducible method for eliciting psychosocial stress (3). However, such paradigms are limited in ecological validity, as they rely on highly structured and context-constrained stressors that only partially reflect real-world stress experiences (4). In this context, virtual reality (VR) has emerged as a promising methodological alternative, enabling stress induction within immersive, interactive, and context-rich environments while maintaining experimental control (5). This technological shift has led to a rapid expansion of VR-based stress paradigms across cognitive neuroscience, psychophysiology, and applied psychology (6).

Recent studies indicate that VR-based paradigms can reliably elicit subjective stress and autonomic activation; however, the extent to which different VR paradigms engage endocrine, neural, and behavioral stress systems depends strongly on the type of stressor employed (7). Social-evaluative VR paradigms, particularly adaptations of the TSST (TSST-VR), consistently induce subjective and autonomic stress responses, whereas HPA-axis activation is more variable and appears to depend on the salience and credibility of social-evaluative cues, such as the interactivity and responsiveness of virtual evaluators (8, 9). These findings are consistent with models emphasizing social evaluation and uncontrollability as primary drivers of cortisol reactivity (10).

In contrast, threat- and fear-based VR paradigms—such as immersive height exposure, horror scenarios, or environments simulating physical danger—tend to elicit strong autonomic and affective responses, particularly in electrodermal activity, often accompanied by less consistent endocrine activation (11). In these paradigms, endocrine responses are frequently weaker or delayed, suggesting preferential engagement of fast-acting sympathetic systems over slower HPA-axis responses (12, 13). This pattern supports the notion of dissociation between stress-response domains and indicates that VR preserves fundamental distinctions between socially evaluative and threat-based stressors observed in non-virtual settings (6).

More recently, VR research has moved beyond discrete stress tasks toward complex, ecologically valid, and dynamically evolving environments in which stressors are embedded within task demands. For example, Tyagi et al. (14) implemented a VR-based emergency response training scenario for firefighters involving unpredictable hazards, time pressure, and high sensory load, where stress emerged from task execution rather than from a discrete manipulation (15). Using functional near-infrared spectroscopy (fNIRS), the authors demonstrated reduced dorsolateral prefrontal cortex activation alongside altered functional connectivity, suggesting compensatory neural mechanisms during learning under stress (14). Such findings highlight the potential of VR to capture stress-related cognitive and neural dynamics in contexts closely approximating real-world environments.

Taken together, these findings suggest that VR does not induce stress as a single, homogeneous construct but rather selectively engages distinct components of the stress response depending on paradigm characteristics (7, 14, 16). This heterogeneity has important implications for both theoretical models of stress and the methodological design of VR-based experiments, as the choice of paradigm reflects implicit assumptions regarding the stress mechanisms under investigation (6).

At the same time, increasing attention has been directed toward individual vulnerability factors, particularly early adversity, in shaping stress reactivity. Developmental research indicates that adverse childhood experiences do not uniformly increase stress sensitivity but instead calibrate stress systems in a context-dependent manner, potentially leading to heightened reactivity to specific classes of stressors, such as social evaluation or threat-related cues (17–19). Despite this, the role of early adversity in VR-based stress paradigms remains insufficiently integrated and systematically examined.

Stress-related alterations in eating behavior represent one of the most clinically relevant behavioral outcomes associated with both acute stress and early adversity. A substantial body of research indicates that stress can modulate appetite, food preferences, and decision-making, often promoting increased consumption of palatable, energy-dense foods. Importantly, individuals with a history of adverse childhood experiences may exhibit heightened vulnerability to such maladaptive eating patterns, including emotional eating and overeating, particularly under conditions of stress. In this context, virtual reality–based stress paradigms offer a unique opportunity to experimentally model how stress interacts with individual vulnerability to influence food-related behaviors within controlled yet ecologically valid environments.

Therefore, the present scoping review aims to: (1) systematically map the diversity of VR-based stress induction paradigms; (2) classify these paradigms based on their underlying stress mechanisms; and (3) examine how different paradigms engage distinct stress-response systems, with particular consideration of early adversity as a moderating factor. Additionally, this review adopts a translational perspective by exploring how these paradigms may serve as experimental models for investigating maladaptive stress-related behaviors, including eating behavior.

## 2 Methods

### 2.1 Design

**Figure 1:**
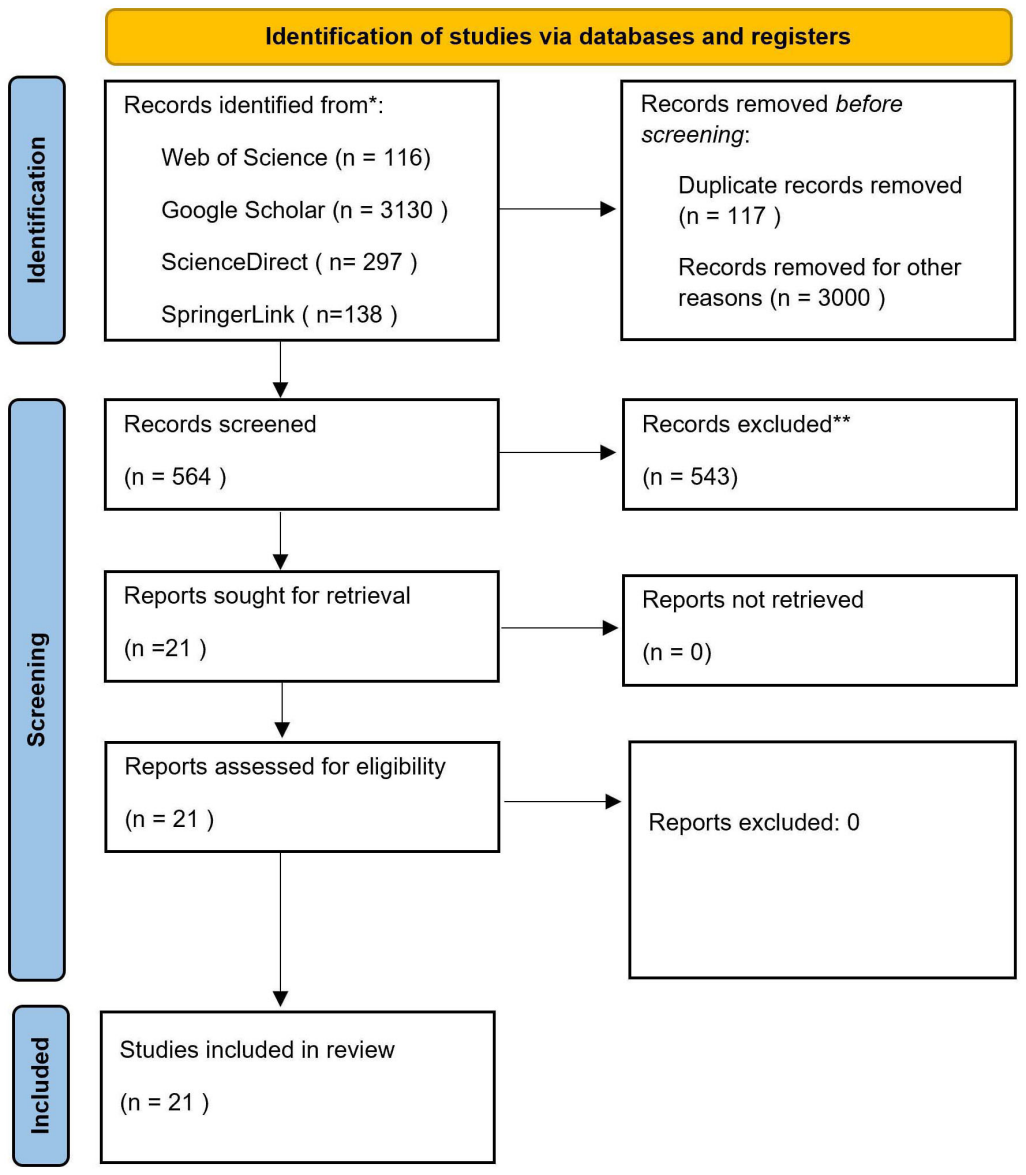
PRISMA 2020 flow diagram illustrating the literature search and study selection process. A total of 3,681 records were identified through database searches. After duplicate removal and screening, 21 studies fulfilled the eligibility criteria and were included in the final review.

A structured literature search, illustrated in **Figure 1**, was conducted in June 2026 using four electronic databases: Web of Science, SpringerLink, ScienceDirect and Google Scholar. The search aimed to identify studies investigating virtual reality (VR)-based stress induction paradigms and their associated stress-response systems, while considering early adversity as a potential moderator of stress reactivity. Importantly, the review was not restricted to a single stress paradigm (e.g., social-evaluative stress), but was designed to capture the full spectrum of VR-based stress induction approaches across different conceptual and methodological frameworks. The search strategy was developed iteratively by the research team, with multiple combinations of keywords tested during the preliminary search phase. To improve methodological sensitivity while maintaining specificity, the search strategy was broadened compared with the initial approach. The following search terms were used: (“virtual reality”) AND (“stress induction paradigm” OR “psychosocial stress”). The search syntax was adapted to the technical requirements of each database while preserving the same conceptual search domains. Broader or alternative keyword combinations tested during pilot searches yielded either excessively heterogeneous results or a high proportion of irrelevant records, reducing feasibility for systematic screening. The Google Scholar search was conducted in accordance with recommendations from the Joanna Briggs Institute (JBI) manual for scoping reviews, which recommended sequential screening until no additional relevant studies are identified. Google Scholar records were screened in order of relevance, and screening was discontinued when consecutive pages yielded no additional eligible studies. This approach was adopted to maximize feasibility while minimizing the inclusion of increasingly irrelevant records generated by the broad indexing strategy of Google Scholar. The literature search included studies published between January 2012 and June 2026. This yielded the following number of records: Web of Science (n = 116), ScienceDirect (n = 297), SpringerLink (n = 138), and Google Scholar (n = 3130). Google Scholar results were screened sequentially, and screening was discontinued when no additional relevant studies were identified and consecutive pages yielded no new relevant records. All retrieved records were exported for further screening, and duplicate entries were removed prior to title and abstract screening. The study selection process is illustrated in **Figure 1**.

Screening was conducted in two stages: (1) title and abstract screening and (2) full-text eligibility assessment. To enhance methodological rigor, the screening process was performed independently by two researchers. Discrepancies were resolved through discussion and consensus. This scoping review included studies that met the following inclusion criteria: (1) use of virtual reality technology; (2) assessment of stress or stress-related mechanisms; and (3) evaluation of the physiological and/or psychological effects of stress. The exclusion criteria comprised studies not published in English and studies for which the full text was not available, including non–open-access publications. Studies for which the full text was unavailable were excluded. We acknowledge that this approach may have introduced selection bias by potentially omitting relevant studies published in subscription-based journals; therefore, this issue is discussed as a limitation of the present review. In addition, a language restriction was applied, excluding studies published in languages other than English. This decision was made to avoid potential interpretation errors in the absence of professional translation resources and to ensure consistency in data extraction. Restricting the review to English-language publications aimed to support the accuracy, transparency, and reproducibility of the review process. Data from the included studies were extracted and synthesized descriptively. Studies were grouped into clusters based on shared conceptual and methodological characteristics of the stress induction paradigms. The five-cluster framework was developed inductively after data extraction and was intended to organize studies according to their predominant stress induction mechanism rather than establish mutually exclusive categories. Studies were assigned to clusters based on the primary stressor employed, the underlying conceptual mechanism, and the principal physiological and psychological response systems investigated. When studies incorporated hybrid paradigms involving more than one stressor type (e.g. combined cognitive and social-evaluative stressors), classification was based on the predominant stress component that constituted the main experimental manipulation. This approach was adopted to improve conceptual clarity while preserving the heterogeneity inherent to VR-based stress induction paradigms. This approach is consistent with scoping review methodology, which aims to map the breadth and structure of a research field rather than to provide quantitative synthesis.

## 3 Results

The included studies were organized into five clusters of virtual reality–based stress paradigms according to shared conceptual, methodological, and stress-induction characteristics. This classification reflects systematic differences in how stress was operationalized across VR environments, including the type of stressor, the underlying mechanisms targeted, and the response systems assessed. The main characteristics of the studies included in this review are summarized in **Table 1**.

**Table 1:** Characteristics of included studies on virtual reality–based stress induction paradigms.

No	Author (Year)	Sample	VR paradigm	Stress type	Core mechanism	Measures	Key findings	Cluster
	Barton et al. (26)	Clinical + controls (N = 140)	Cyberball social exclusion	Social-evaluative	Social evaluation	Behavioral, CTQ	Reduced prosocial behavior linked to childhood maltreatment	1
	Riem et al. (9)	Female students (N = 180)	TSST-VR + social support	Social-evaluative	Social evaluation	Cortisol, HRV, anxiety	Oxytocin improved stress recovery, moderated by ACEs	1
	Kothgassner et al. (6)	Healthy adults	VR Cyberball + TSST-VR	Social-evaluative	Social evaluation	Cortisol, VAS	VR paradigms induced subjective and endocrine stress	1
	Delahaye et al. (24)	Healthy adults (N = 31)	TSST-VR + VR maze	Social-evaluative	Social evaluation	HR, subjective	Stress impaired learning but enhanced flexibility	1
	Liu & Zhang (23)	Students (N = 57)	TSST-VR vs placebo	Social-evaluative	Social evaluation	EDA, HRV, VAS	Sex differences in autonomic stress reactivity	1
	Shiban et al. (22)	Healthy men (N = 45)	TSST (VR vs *in vivo*)	Social-evaluative	Social evaluation	Cortisol, HR, SCL	Reduced cortisol response in VR vs *in vivo*	1
	Montero-López et al. (21)	Students (N = 45)	TSST-VR (HMD vs screen)	Social-evaluative	Social evaluation	Cortisol, SC	Both formats induced stress; modality affected SC	1
	Veling et al. (27)	Mixed clinical sample (N = 170)	VR café social stress	Social-evaluative	Social evaluation	SUD, CTQ	Trauma linked to increased stress reactivity	1
	Zimmer et al. (8)	Healthy men (N = 87)	Refined TSST-VR	Social-evaluative	Social evaluation	Cortisol, HR	VR induced cortisol comparable to *in vivo* TSST	1
	Zimmer et al. (20)	Healthy men (N = 49)	TSST-VR (context manipulation)	Social-evaluative	Social evaluation	Cortisol, HR	Environmental realism did not affect stress	1
	Kerous et al. (29)	Healthy adults (N = 16)	VR Stroop + social stress	Cognitive/social	Cognitive overload	EDA, HRV	EDA sensitive to combined stressors	2
	Gradl et al. (28)	Healthy adults (N = 32)	VR Stroop Room	Cognitive	Cognitive overload	HR, EDA, cortisol	Strong autonomic stress, limited endocrine response	2
	Boccignone et al. (13)	Healthy adults (N = 33)	Height exposure VR	Threat/fear	Threat/fear exposure	EDA, HRV	Strong autonomic fear responses	3
	El Basbasse et al. (30)	Healthy adults (N = 75)	VR plank + EEG	Threat/fear	Threat/fear exposure	EEG, subjective	Fear linked to neural asymmetry patterns	3
	Hanshans et al. (31)	Healthy adults (N = 20)	VR horror	Threat/fear	Threat/fear exposure	EEG, SCR	Reliable acute stress via multimodal measures	3
	Martens et al. (12)	Healthy men (N = 28)	VR height exposure	Threat/fear	Threat/fear exposure	Cortisol, HR	Strong autonomic + delayed cortisol response	3
	Heffer et al. (32)	Healthy adults (N = 55)	VR car accident	Trauma-analogue	Trauma analogue exposure	Intrusions, subjective	Multisensory processing predicts intrusions	4
	Dibbets (33)	Healthy adults (N = 82)	VR train crash	Trauma-analogue	Trauma analogue exposure	HR, intrusions	Emotional response predicts avoidance and intrusions	4
	Tyagi et al. (14)	Firefighters (N = 40)	VR emergency training	Complex/applied	Complex applied stress	fNIRS, performance	Stress reduced PFC activity, altered learning	5
	Lin et al. (34)	Healthy adults (N = 9)	VR metro fire	Complex/applied	Complex applied stress	HR, BP	VR induced physiological stress responses	5
	Varshney et al. (35)	Healthy adults (N = 48)	VR navigation stress	Complex/applied	Complex applied stress	Behavior, stress ratings	Stress shifted navigation to habitual strategies	5

Rather than treating stress as a homogeneous construct, the identified clusters capture distinct approaches to stress induction in VR, ranging from socially mediated and cognitively driven challenges to threat-based, trauma-analogue, and ecologically complex operational contexts. This framework allows for a structured comparison of how different VR paradigms selectively engage cognitive, affective, physiological, and behavioral responses.

Cluster 1 comprises social-evaluative paradigms, in which stress is induced through interpersonal threat, social evaluation, or social exclusion within virtual environments. Cluster 2 includes cognitive and performance-based paradigms, where stress is primarily driven by cognitive load, task difficulty, time pressure, and performance demands. In these paradigms, VR is used to embed cognitively demanding tasks, such as Stroop interference, within immersive environments. Cluster 3 encompasses threat- and fear-based paradigms, in which stress is induced through exposure to scenarios involving perceived physical danger. Cluster 4 consists of analogue trauma paradigms, designed to simulate highly aversive events and examine immediate and short-term cognitive, emotional, and behavioral outcomes. Finally, Cluster 5 includes complex, ecologically valid paradigms that embed stress within dynamic, task-relevant environments, often simulating real-world operational or emergency contexts.

The following sections provide a detailed analysis of each cluster, focusing on their defining features and the stress-response systems they engage.

### 3.1 Cluster 1: social-evaluative virtual reality stress paradigms (TSST-VR and avatar-based social threat)

This cluster includes VR paradigms derived from the Trier Social Stress Test (TSST), which reproduce public speaking and mental arithmetic under evaluative conditions (8, 9, 20–24), as well as social exclusion paradigms such as Cyberball (25, 26) and socially threatening virtual environments (e.g., hostile or crowded social scenes) (27). Across these paradigms, stress induction relies on two core psychosocial components: uncontrollability and social evaluation or rejection.

A substantial proportion of studies implemented TSST-VR variants in which participants delivered a speech (often framed as a mock job interview) and performed mental arithmetic in front of virtual evaluators (8, 9, 20–23). While the fundamental TSST structure remained consistent, studies varied in the level of interactivity and contextual manipulation. Some focused on presentation format, such as head-mounted displays versus large-screen projection (21), whereas others compared *in vivo* and VR implementations of the TSST (8, 22). Additional variations included manipulation of environmental realism (20) and the incorporation of interactive features such as eye-tracking–based feedback to enhance social-evaluative salience (8).

Beyond TSST-based paradigms, several studies emphasized interpersonal stress through social exclusion or adverse social interaction. Kothgassner et al. (25) developed avatar-based analogues of both Cyberball and TSST-VR, demonstrating that multiple psychosocial stress paradigms can be implemented in VR under controlled conditions. Barton et al. (26) employed partial ostracism in a Cyberball paradigm to examine social repair behavior, while Veling et al. (27) used a virtual café environment in which social stressors such as crowding, hostile facial expressions, and out-group avatars were systematically manipulated. These approaches extend stress induction beyond discrete tasks, conceptualizing social threat as an environmental property.

Across this cluster, subjective stress responses were consistently reported and typically increased during or immediately after exposure (8, 20, 22, 23, 27). Physiological responses were more heterogeneous. Several studies assessed endocrine activation through salivary cortisol (8, 9, 20–22, 25), often alongside autonomic measures such as heart rate, skin conductance, or salivary alpha-amylase (8, 20–23). Other studies focused primarily on subjective or autonomic indices (23, 24, 27) or examined behavioral responses following social exclusion (26).

A key finding across TSST-based studies is the dissociation between stress-response domains. Autonomic and subjective responses were generally robust across VR implementations, whereas HPA-axis activation was more sensitive to paradigm design. Shiban et al. (22) reported comparable subjective and autonomic responses in VR and *in vivo* TSST conditions but lower cortisol responder rates in VR, indicating reduced endocrine activation. In contrast, Zimmer et al. (8)demonstrated that a refined TSST-VR incorporating enhanced social-evaluative cues could elicit cortisol responses comparable to *in vivo* TSST. Additionally, Zimmer, Wu, and Domes (20) found that replicating the real laboratory environment in VR did not significantly influence stress responses or perceived presence, suggesting that social-evaluative structure, rather than environmental realism, is the critical determinant of stress induction.

Several studies in this cluster also examined moderators of stress reactivity, including sex, early adversity, and clinical vulnerability. Liu and Zhang (23) reported sex differences in autonomic reactivity using a TSST-VR compared with a placebo VR condition, highlighting differential physiological responses between men and women. Other studies either reported sex-related differences in perceived presence (21) or were restricted to male samples, limiting generalizability (8, 20, 22).

Early adversity emerged as a relevant moderator across multiple paradigms. Riem et al. (9) demonstrated that negative childhood experiences influenced anxiety and cortisol recovery in a TSST-VR paradigm involving oxytocin administration and social support. Similarly, Veling et al. (27) reported that childhood trauma was associated with increased subjective distress and stress reactivity in socially threatening VR environments, with effects amplified by the number of social stressors. Barton et al. (26) further showed that childhood maltreatment, particularly emotional neglect, was associated with reduced prosocial behavior following social exclusion.

Clinical and liability-related factors were most directly examined by Veling et al. (27), who found that individuals with higher psychosis liability exhibited heightened stress responses in socially threatening VR contexts. These findings extend the relevance of this cluster beyond healthy populations and highlight its potential for translational research.

Overall, social-evaluative VR paradigms consistently reproduce key features of psychosocial stress, including uncontrollability and social evaluation or rejection, and reliably elicit subjective and autonomic responses. However, endocrine activation appears more dependent on the specific implementation of social-evaluative cues. Importantly, individual differences, particularly early adversity, sex, and clinical vulnerability, act as moderators of stress reactivity within these paradigms, underscoring their relevance for investigating vulnerability-related mechanisms in stress research.

### 3.2 Cluster 2: cognitive and performance-based virtual reality stress paradigms

Across the studies included in this cluster, stress induction was grounded in task-related cognitive interference rather than interpersonal evaluation. The Stroop task served as the primary stressor, relying on response conflict and attentional control demands to elicit stress (28, 29). In these paradigms, VR functioned not merely as a display medium but as a means to amplify cognitive load through environmental manipulation.

In the Stroop Room, cognitive stress was systematically manipulated across multiple difficulty levels by introducing time pressure, environmental constriction (e.g., walls closing in), and additional attentional demands (28). These manipulations progressively increased task difficulty within an immersive setting. In contrast, Kerous et al. (29) embedded the Stroop task within navigable virtual environments, such as a park or a hospital office, emphasizing interaction and sensorimotor engagement through the use of virtual hands. While cognitive load remained the primary stressor in both studies, Gradl et al. (28) additionally contrasted pure cognitive stress, social scrutiny, and their combination, enabling dissociation of stressor types within a single VR protocol.

Both studies relied primarily on autonomic and physiological markers to quantify stress responses, consistent with the performance-based nature of the paradigms. Electrodermal activity (EDA), heart rate (HR), and heart rate variability (HRV) were central measures, and both studies demonstrated that cognitively demanding VR tasks reliably elicited autonomic activation. Gradl et al. (28) reported substantial increases in HR and EDA, alongside reductions in HRV indices, reflecting sympathetic dominance under high cognitive load. Similarly, Kerous et al. (29) observed autonomic changes during VR Stroop performance; however, their factorial design revealed differential sensitivity across physiological measures. Specifically, EDA was most responsive when cognitive load was combined with social scrutiny, whereas HR and HRV were more strongly modulated by cognitive load alone. These findings suggest that different physiological indices capture distinct aspects of stress within cognitively driven VR paradigms.

The inclusion of endocrine measures differed between studies. Gradl et al. (28) incorporated salivary cortisol and alpha-amylase alongside autonomic indices, reporting endocrine changes under specific high-demand conditions, although these were less consistent than autonomic responses. In contrast, Kerous et al. (29) focused exclusively on autonomic outcomes, reflecting an emphasis on immediate physiological reactivity during task execution rather than HPA-axis activation.

Overall, cognitive and performance-based VR paradigms induce stress primarily through increased task demands and attentional load, leading to robust autonomic activation and performance-related effects, while endocrine responses appear less consistently engaged. These paradigms therefore capture stress as a function of cognitive overload and performance pressure rather than social or emotional threat.

### 3.3 Cluster 3: threat- and fear-based virtual reality stress paradigms

Across studies, stress induction was achieved through immersive scenarios involving perceived physical danger, without requiring social evaluation or task performance. These paradigms primarily relied on emotional salience and fear as the core stress-induction mechanisms. Height-based exposure paradigms were commonly used, including elevator ascent followed by exposure to a narrow platform or plank at extreme altitude (12, 13). Similarly, El Basbasse et al. (30) implemented a virtual plank paradigm contrasting a high-altitude condition with a neutral street-level environment to isolate fear-driven stress. In addition, Hanshans et al. (31) extended this approach by using immersive 180° horror stimuli, eliciting fear through threatening audiovisual content rather than spatial height. Despite differences in stimulus format, all paradigms were characterized by the absence of social-evaluative threat or performance demands, distinguishing them clearly from social and cognitive stress models.

A defining feature of this cluster is the strong emphasis on physiological and neural markers of acute stress. Electrodermal activity (EDA) emerged as a particularly sensitive indicator of fear-induced stress. Height-based paradigms showed sustained increases in EDA that differentiated fearful from non-fearful participants (13), while immersive horror paradigms demonstrated large skin conductance responses during exposure (31).

Cardiovascular measures consistently indicated robust autonomic activation. Height exposure and plank paradigms were associated with increases in heart rate, blood pressure, and changes in heart rate variability, reflecting sympathetic dominance (12, 13). Beyond peripheral physiology, neural measures further supported the sensitivity of these paradigms. Mobile electroencephalography (EEG) recordings revealed patterns such as frontal alpha asymmetry and increased beta activity associated with negative affect and fear, highlighting neural oscillatory dynamics as complementary markers of threat-based stress (30, 31).

Endocrine responses were assessed less consistently across studies. In one study, VR height exposure was associated with maintenance of salivary cortisol levels relative to the expected diurnal decline, suggesting a distinct pattern of HPA-axis engagement compared with social-evaluative paradigms. Subjective ratings of fear, anxiety, and stress closely paralleled physiological responses, with strong associations observed between experienced fear and autonomic reactivity across immersive conditions (12, 13, 30, 31).

Overall, threat- and fear-based VR paradigms reliably induce acute, emotion driven stress through sensory immersion. These paradigms are characterized by robust autonomic activation and complementary neural responses, while endocrine activation appears more variable. This pattern highlights the capacity of VR to model ecologically valid emotional stress and reinforces the distinction between fear-based and social-evaluative stress mechanisms.

### 3.4 Cluster 4: virtual reality analogue and adverse event paradigms

Studies in this cluster implemented realistic accident simulations presented from a non-evaluative perspective, inducing stress through exposure to aversive events rather than through social threat or performance demands. One study employed a virtual car accident experienced from a passenger’s perspective (32), whereas another developed a train crash scenario viewed from a bystander perspective (33). In both cases, VR exposure elicited immediate stress responses, reflected in increased subjective distress and negative affect, as well as physiological changes such as elevated heart rate and reduced heart rate variability (32, 33). These findings support the validity of immersive VR as an experimental analogue for inducing acute trauma-related stress under controlled conditions.

A defining feature of this cluster is the systematic assessment of post-exposure outcomes over time, extending beyond immediate stress responses. Both studies employed intrusion diaries over a seven-day period following VR exposure, allowing prospective tracking of the frequency and distress associated with intrusive memories (32, 33). This longitudinal approach aligns VR-based paradigms with established analogue trauma research while enhancing ecological validity. In addition, Dibbets (33) assessed trauma-related cognitions and directly quantified behavioral avoidance using a follow-up VR task, in which participants’ scene selections served as an objective measure of posttraumatic avoidance.

Beyond documenting post-event symptoms, both studies examined mechanisms underlying individual differences in trauma-related outcomes. Heffer et al. (32) introduced a multisensory emotion processing framework, demonstrating that individual differences in audiovisual emotion integration predicted subsequent intrusive memories. Greater multisensory facilitation for threat-related cues was associated with higher intrusion frequency, whereas greater cross-modal interference predicted fewer and less distressing intrusions. These findings suggest that sensory–cognitive processing styles may represent mechanisms linking acute stress exposure to later intrusive symptomatology. In contrast, Dibbets (33) focused on peri-traumatic emotional responses and trait-level risk factors, identifying negative emotional responses during VR exposure, trait anxiety, and avoidant coping as predictors of intrusive memories and avoidance behavior.

Although both studies share a common conceptual framework, they differ in their emphasis on outcome domains. Heffer et al. (32) highlighted perceptual and cognitive predictors of intrusions through multisensory processing and longitudinal symptom tracking, whereas Dibbets (33) adopted a broader approach encompassing physiological reactivity, cognitive appraisals, and behavioral avoidance.

Overall, analogue trauma VR paradigms connect experimental stress induction with trauma research by integrating ecologically valid simulations and the prospective assessment of intrusions, avoidance, and trauma-related cognitions. These paradigms capture the progression from stress exposure to early posttraumatic cognitive and behavioral outcomes, going beyond immediate physiological responses.

### 3.5 Cluster 5: complex ecologically valid virtual reality stress paradigms

This cluster includes VR paradigms that embed stress within complex, dynamic, and task-relevant environments designed to approximate real-world conditions. Unlike discrete stress-induction tasks, these paradigms integrate stressors directly into ongoing activities, such as emergency response, evacuation, or navigation under uncertainty.

Tyagi et al. (14) implemented a VR-based emergency response training scenario for firefighters within a chemical plant shutdown context. Stress was induced through unpredictable hazards, including explosions, smoke, alarms, and time pressure, requiring participants to acquire and apply procedural knowledge under acute stress. Similarly, Lin et al. (34) examined passenger evacuation during a simulated metro fire, emphasizing sensory realism through exposure to fire, smoke, alarms, and task-related demands. While grounded in emergency simulation, this paradigm targeted civilian stress responses rather than professional training. In addition, Varshney et al. (35) developed a large-scale immersive navigation task in which stress was induced through blocked paths, reduced visibility, threatening auditory cues, and strict time constraints during wayfinding. Although not explicitly framed as an emergency scenario, this paradigm captured situational uncertainty and environmental threat analogous to real-world navigation challenges.

Together, these studies reflect a continuum of ecological validity, ranging from highly realistic emergency scenarios to more simplified yet functionally comparable stress-inducing contexts. Across paradigms, stress arises as an inherent feature of task engagement rather than as an externally imposed manipulation.

A key characteristic of this cluster is the heterogeneity of outcome measures, reflecting the applied nature of these paradigms. Tyagi et al. (14) integrated behavioral performance metrics, self-reported anxiety, and neural dynamics, whereas Lin et al. (34) primarily assessed physiological markers such as heart rate and blood pressure. Varshney et al. (35) focused on behavioral strategies and individual differences in navigation performance. Across studies, stress consistently influenced behavior and task performance, although the direction and magnitude of effects varied.

Notably, Tyagi et al. (14) reported that stress was associated with slower learning rates and reduced dorsolateral prefrontal cortex activation, accompanied by increased functional connectivity within prefrontal networks and between prefrontal and premotor regions, suggesting compensatory neural mechanisms during learning under stress. In addition, both Lin et al. (34) and Varshney et al. (35) indicated that stress-related behavioral responses were moderated by individual differences in psychological characteristics.

Overall, complex VR paradigms demonstrate that stress can be embedded within ecologically valid, goal-directed environments, where it directly shapes behavior, learning, and decision-making. These models extend beyond traditional stress induction by capturing dynamic interactions between stress, cognition, and performance in contexts that closely resemble real-world demands.

## 4 Discussion

This scoping review highlights the multidimensional and context-dependent nature of stress as elicited through virtual reality (VR) paradigms. Rather than representing a unitary construct, stress responses in VR environments emerge through the differential engagement of subjective, autonomic, endocrine, and behavioral systems, shaped by both paradigm characteristics and individual vulnerability factors. The findings further suggest that VR paradigms are not theoretically neutral; instead, they embed implicit assumptions about what constitutes a stress-relevant process, thereby influencing both the type of responses elicited and their interpretation.

A central contribution of this review is the integration of VR-based stress paradigms with a developmental vulnerability framework centered on early adversity. An additional consideration concerns the construct validity of VR-based stress paradigms. Although all paradigms were examined within the broader framework of stress induction, they do not necessarily assess the same underlying construct. Social-evaluative paradigms primarily elicit psychosocial stress, threat-based paradigms overlap substantially with fear and anxiety responses, trauma analogue paradigms capture reactions to aversive events, and complex simulation paradigms frequently involve cognitive workload, uncertainty, and performance demands. Therefore, these paradigms should not be considered interchangeable measures of a unitary stress construct. Rather, they represent distinct but partially overlapping dimensions of stress-related experiences. This distinction may help explain the variability observed across subjective, autonomic, endocrine, neural, and behavioral responses and highlights the importance of selecting VR paradigms according to the specific research question being addressed. Rather than treating VR stress paradigms as purely methodological tools, the present findings suggest that different paradigm types may differentially capture stress-relevant processes depending on individuals’ developmental histories; however, these interpretations are primarily based on a limited number of studies directly assessing adverse childhood experiences. Importantly, incorporating early adversity provides a critical framework for understanding variability in stress reactivity, emphasizing that stress induction in VR is not only paradigm-dependent but also person-specific. Within this context, behavioral outcomes emerge as a central yet underutilized dimension, offering unique insights into how stress translates into real-world relevant actions and adaptations. Together, these observations underscore the need for a more integrative and theoretically aligned approach to VR-based stress research, in which paradigm selection, individual history, and multidomain assessment are considered in conjunction.

Dissociation of Stress-Response Domains in VR Paradigms.

Across VR-based stress paradigms, stress responses are not unitary but differentially engaged, with subjective, autonomic, and hypothalamic–pituitary–adrenal (HPA) axis components varying as a function of stressor type and virtual environment design.

Social-evaluative paradigms derived from the Trier Social Stress Test (TSST) consistently elicit strong subjective stress responses and robust autonomic activation, reflected in increases in heart rate, electrodermal activity, and self-reported stress or anxiety (8, 21, 22, 34). However, engagement of the HPA axis within TSST-VR paradigms is more variable. Direct comparisons between *in vivo* and VR-based TSST implementations indicate that cortisol responses are often attenuated in VR (8, 22, 36). This pattern suggests that social evaluation alone may be sufficient to activate autonomic stress systems, whereas robust HPA-axis activation depends on additional features such as social salience, credibility, and interactivity.

In contrast, threat- and fear-based VR paradigms exhibit a distinct response profile characterized by strong and consistent electrodermal activation, closely aligned with subjective fear and arousal, while endocrine responses remain more variable and temporally dissociated from those observed in social-evaluative stress (12, 13, 31). These findings highlight the preferential engagement of fast-acting autonomic systems in fear-driven contexts.

Importantly, behavioral outcomes do not always align with autonomic and endocrine responses. Several studies demonstrate that stress-related changes in behavior—such as alterations in social interaction, learning strategies, or decision-making—are not fully explained by concurrent physiological markers (13, 32, 33, 35). For example, social exclusion paradigms reveal changes in social behavior following stress exposure, which may occur even in the absence of strong physiological responses and are linked to early adversity (26). Similarly, complex VR environments show stress-related shifts in navigation strategies, learning efficiency, and decision-making processes that cannot be reduced to autonomic or endocrine activation alone (14, 35).

Early adversity may further accentuate this dissociation between stress-response systems. Studies incorporating adverse childhood experiences suggest that early adversity is more consistently associated with heightened subjective distress, altered autonomic reactivity, and stress-related behavioral changes than with HPA-axis activation (9, 26, 27, 33).

Taken together, these findings indicate that VR does not induce stress as a singular, homogeneous construct. Instead, VR paradigms selectively engage distinct components of the stress response in a stressor-dependent manner, shaped by both environmental design and individual vulnerability factors.

Conceptualizing Stress in Virtual Reality: Paradigm-Dependent Mechanisms.

Existing literature highlights that the selection of a stress paradigm fundamentally shapes what is considered a stress-relevant process. Different VR paradigms preferentially engage distinct psychological and neurobiological mechanisms (37). Consequently, the choice of a VR stress paradigm extends beyond methodological considerations, influencing both theoretical interpretation and practical application (38, 39).

Social-evaluative VR paradigms, particularly TSST-VR, are grounded in models that conceptualize stress as arising from social judgment, uncontrollability, and threats to the social self. Within this framework, stress relevance is defined by perceived evaluation and self-conscious emotions such as shame, embarrassment, or fear of negative evaluation (8, 21–24).

In contrast, threat- and fear-based VR paradigms are based on models that conceptualize stress as an adaptive response to perceived physical danger or environmental threat. In these paradigms, stress is primarily induced through sensory salience and emotional arousal rather than social evaluation or performance demands (13, 31).

Cognitive and performance-based VR paradigms, such as immersive Stroop or multitasking environments, conceptualize stress in terms of demands on executive control, attentional regulation, and time pressure (28, 29). Here, stress relevance is defined by cognitive load and performance monitoring rather than emotional or social threat (37). These paradigms are aligned with neurocognitive models linking stress to impairments in prefrontal cortex functioning (40, 41). Consistent with this framework, they predominantly elicit autonomic responses and performance-related effects, while HPA-axis activation remains inconsistent or limited (30, 35).

Virtual reality analogue trauma paradigms adopt a different theoretical perspective, conceptualizing stress as an acute aversive event with downstream consequences for memory, cognition, and behavior (32). In these models, stress relevance extends beyond immediate physiological responses and is reflected in post-exposure outcomes such as intrusive memories, avoidance behavior, and maladaptive cognitions. Recent VR studies emphasize prospective assessment of these outcomes (25, 33), positioning stress as a dynamic process unfolding over time rather than a transient physiological state (42, 43).

Taken together, these observations indicate that VR stress paradigms embed implicit theoretical assumptions about the nature of stress and the mechanisms through which it operates. Therefore, paradigm selection should be guided by the specific stress system or outcome of interest rather than by immersion alone. Such alignment is essential for precise hypothesis testing, improved comparability across studies, and cumulative theory development in VR-based stress research.

Early Adversity as a Determinant of Stress Relevance Across Virtual Reality Paradigms.

Although early adversity emerges as a relevant theme throughout this review, only a subset of the included studies directly assessed adverse childhood experiences or childhood trauma. Therefore, adversity-specific conclusions should be interpreted with caution. The available evidence suggests that early adversity may influence physiological, psychological, and behavioral responses elicited by VR-based stress paradigms; however, this relationship remains underexplored and cannot yet be generalized across all categories of VR stress induction. Consequently, the role of early adversity should currently be regarded as a promising avenue for future research rather than an established finding across the entire field.

Integrating early adversity and adverse childhood experiences (ACEs) into the interpretation of VR-based stress paradigms provides a framework for understanding why different paradigms elicit divergent stress responses across individuals. A substantial body of research indicates that early adversity calibrates stress systems in a domain-specific and context-dependent manner (17–19). From this perspective, early adversity does not uniformly increase stress reactivity but instead selectively sensitizes individuals to particular classes of threat.

Applied to VR-based stress research, this framework shifts the focus from whether a paradigm induces stress to for whom it is stressful and through which response systems (44). Social-evaluative VR paradigms, such as TSST-VR and socially threatening virtual environments, may be particularly salient for individuals with histories of interpersonal adversity, emotional neglect, or social rejection (31, 33). These forms of early adversity are associated with heightened sensitivity to social evaluation, rejection, and negative self-referential processing, as well as altered regulation of autonomic and HPA-axis responses in social contexts (17, 45). Consistent with this view, VR studies incorporating ACEs report amplified subjective stress, altered recovery dynamics, and stress-related behavioral changes in individuals with higher levels of childhood trauma, even when physiological responses are not uniformly elevated (27, 33, 46).

Threat- and fear-based VR paradigms may differentially affect individuals exposed to threat-related adversity, such as violence, unpredictability, or chronic danger (47, 48). Developmental models differentiate between threat and deprivation, with threat-related adversity associated with increased neural and autonomic reactivity to fear- and danger-related cues (47). Accordingly, immersive VR scenarios involving height exposure, horror stimuli, or environmental danger may be particularly stress-relevant for individuals with threat-related ACE profiles, potentially eliciting exaggerated autonomic activation, sustained vigilance, or avoidance behavior (13, 31). However, most threat-based VR studies have not systematically assessed early adversity, leaving these theoretically important individual differences largely unexplored.

Cognitive and performance-based VR paradigms represent an additional pathway through which early adversity may shape stress reactivity (49). Early adversity has been associated with long-term alterations in executive functioning, cognitive flexibility, and emotion regulation, particularly under conditions of high demand and uncertainty (50). In this context, cognitively demanding VR tasks may induce stress in adversity-exposed individuals through mechanisms of overload or perceived loss of control rather than emotional threat (51). These findings suggest that cognitive VR paradigms may be especially relevant for individuals with adversity-related vulnerabilities (52).

Overall, early adversity may serve as a central organizing factor linking VR stress paradigm type to individual vulnerability profiles. Aligning paradigm selection with developmental history—such as threat-related, social, or cognitive adversity—may improve the precision with which stress relevance and response patterns are assessed across individuals.

Behavioral Outcomes as a Central but Underutilized Dimension of Stress in Virtual Reality.

Despite substantial advances in physiological and neurobiological measurement, behavioral outcomes remain underexplored in VR-based stress research. This stands in contrast to theoretical models of stress that position behavior as a central component of the stress response, reflecting how individuals act under conditions of threat, uncertainty, or overload (14, 35, 53).

VR-based paradigms are uniquely suited to capture behavioral adaptation, as they embed stressors within interactive, goal-directed environments while maintaining experimental control (16, 25). Unlike traditional laboratory paradigms, which often separate stress induction from subsequent behavioral assessment, VR enables continuous measurement of behavior during stress exposure. This allows for context-dependent interpretation of stress responses rather than *post hoc* inference of their consequences (33).

The present scoping review highlights the added value of behavioral outcomes across different VR stress paradigms. In social-evaluative and interpersonal contexts, behavioral responses reveal stress-related effects that are not fully explained by physiological reactivity (26, 35). For instance, Barton et al. (26) showed that social exclusion in a virtual Cyberball paradigm altered prosocial repair behavior, with higher levels of childhood maltreatment predicting reduced affiliative responses. Notably, these behavioral differences emerged even in the absence of strong physiological mediation, suggesting that behavioral adaptation may reflect developmental vulnerability pathways distinct from autonomic or HPA-axis activation (22, 44).

Similar dissociations between behavioral and physiological responses have been observed in more complex VR environments. Varshney et al. (35) demonstrated that stress during immersive navigation tasks altered decision-making strategies, with participants under stress favoring habitual routes over flexible exploration and showing reduced shortcut use. These effects were moderated by individual differences in spatial anxiety and baseline spatial knowledge, consistent with evidence that stress promotes reliance on habitual control, particularly in vulnerable individuals (54).

Behavioral outcomes are especially central in analogue trauma paradigms, where post-exposure cognition and behavior constitute primary endpoints rather than secondary correlates. Heffer et al. (32) demonstrated that individual differences in multisensory processing of threat-related cues during VR exposure predicted the frequency and distress of intrusive memories over the following week, linking perceptual–cognitive processing under stress to downstream behavioral and experiential outcomes.

Taken together, these findings underscore the importance of systematically incorporating behavioral measures into VR-based stress research. VR provides a unique opportunity to assess avoidance, decision-making under uncertainty, coping strategies, and performance under stress in ecologically valid contexts. Integrating behavioral outcomes with physiological and subjective measures may therefore offer a more comprehensive understanding of stress as a multidimensional process.

In a larger context, current evidence indicates that VR does not induce stress as a single, homogeneous construct but rather selectively engages distinct components of the stress response depending on paradigm characteristics (7, 14, 16). This heterogeneity has important implications for both theoretical models of stress and the methodological design of VR-based experiments, as the choice of paradigm reflects implicit assumptions regarding the stress mechanisms under investigation (6).

Of particular importance, VR-based stress paradigms can serve as flexible experimental platforms for modeling how early adversity shapes stress reactivity across multiple response domains. This framework provides a foundation for future research aimed at investigating maladaptive stress-related behaviors, including those relevant to psychiatric and behavioral conditions.

Within this framework, eating behavior represents a particularly relevant yet underexplored behavioral domain in VR-based stress research. Stress is known to influence food-related decision-making, reward sensitivity, and self-regulation, processes that can be directly assessed in immersive virtual environments. VR paradigms allow for the integration of realistic food cues and eating contexts, enabling the investigation of stress-induced changes in food choice, portion selection, and consumption behavior in real time. Importantly, these effects may be amplified or qualitatively altered in individuals with early adversity, for whom stress reactivity is often shaped by developmental experiences. Thus, integrating food-related behavioral outcomes into VR stress paradigms may provide a valuable avenue for examining mechanisms underlying maladaptive eating patterns.

However, none of the included studies directly assessed food choice, appetite, eating behavior or other nutrition-related outcomes. Therefore, these translational implications should be interpreted as potential future applications rather than findings directly supported by the present review.

### 4.1 Limitations

This review has several limitations that should be considered when interpreting the findings. First, the number of included studies was relatively small, limiting the breadth of evidence available for certain VR stress paradigms. Second, substantial heterogeneity was observed across studies regarding stress induction procedures, outcome measures, and the physiological and psychological domains assessed, limiting direct comparability between paradigms.

Third, the review was restricted to English-language studies and to studies with accessible full texts, which may have introduced selection bias by underrepresenting relevant evidence published in subscription-based journals or other languages. Fourth, only a subset of the included studies directly assessed adverse childhood experiences or childhood trauma, limiting the strength of conclusions regarding the role of early adversity across VR-based stress paradigms.

In addition, no formal quality assessment or risk-of-bias appraisal was conducted, as the review followed a scoping review methodology focused on mapping the available evidence rather than evaluating study quality. Similarly, no quantitative synthesis or meta-analysis was performed due to the considerable methodological heterogeneity across studies.

Finally, the generalizability of the findings is limited because many studies included relatively small samples and predominantly involved healthy adults, university students, or male-only populations. Therefore, caution is warranted when extrapolating these findings to broader or more clinically diverse populations.

## 5 Conclusions

This scoping review demonstrates that VR does not induce stress as a uniform construct, but rather selectively engages distinct components of the stress response such as subjective, autonomic, endocrine, neural and behavioral stress depending on the type of paradigm employed. Socio-evaluative, threat-based, cognitive, analogue trauma and ecologically complex VR paradigms each operationalize stress through different mechanisms and therefore activate partially dissociable stress systems.

Moreover, integrating the role of early adversity within VR-based stress paradigms represents a promising and still underexplored avenue for research. Although relatively few studies have directly investigated ACE profile shape stress reactivity in virtual environments, emerging evidence suggests that developmental history may meaningfully influence how individuals perceive and respond to different classes of VR stressors. Given that only a limited number of studies directly assessed adverse childhood experiences, these associations should currently be considered exploratory rather than established. Rather than assuming uniform stress induction effects, future research could systematically evaluate whether specific ACE patterns are associated with differential subjective, autonomic, endocrine, or behavioral responses across social-evaluative, threat-based, or cognitive VR paradigms. Exploring this link would not only deepen our understanding of individual variability in stress relevance, but also help clarify whether VR paradigms can serve as sensitive tools for detecting adversity-related vulnerabilities in stress regulation.

From a translational perspective, the integration of VR-based stress paradigms with the study of eating behavior represents a promising direction for future research. By enabling the simultaneous assessment of stress responses and food-related behaviors within ecologically valid environments, VR offers a powerful platform for investigating mechanisms underlying stress-related eating, particularly in individuals with early adversity. Such approaches may contribute to the future development of more personalized and context-sensitive models of nutrition and mental health; however, these applications remain speculative and require dedicated empirical investigation before they can be considered evidence-based approaches within the precision and personalized nutrition.
